# Lung Damage Induced by *Plasmodium berghei* ANKA in Murine Model of Malarial Infection is Mitigated by Dietary Supplementation with DHA-Rich Omega-3

**DOI:** 10.1021/acsinfecdis.4c00482

**Published:** 2024-09-20

**Authors:** Carolina David-Vieira, Barbara Albuquerque Carpinter, Jéssica Correia Bezerra-Bellei, Letícia Ferreira Machado, Felipe Oliveira Raimundo, Cinthia Magalhães Rodolphi, Daniela Chaves Renhe, Isabella Rodrigues Nogueira Guedes, Fernanda Mikaela Moreira Gonçalves, Ludmila Ponce Monken Custódio Pereira, Marcos Vinicius Rangel Ferreira, Haroldo Lobo dos Santos Nascimento, Adolfo Firmino Neto, Flávia Lima Ribeiro Gomes, Vinicius Novaes Rocha, Juciane Maria de Andrade Castro, Kézia Katiani Gorza Scopel

**Affiliations:** † Research Centre of Parasitology, Department of Parasitology, Microbiology and Immunology and Post-Graduate Program in Biological Science, Federal University of Juiz de Fora, Juiz de Fora 36036-900, Brazil; ‡ Laboratory of Malaria Research, Oswaldo Cruz Institute, Rio de Janeiro, Fiocruz 21040-360, Brazil; § Research Centre of Pathology and Veterinary Histology, Department of Veterinary Medicine, Federal University of Juiz de Fora, Juiz de Fora 36036-900, Brazil

**Keywords:** malaria, severe malaria, ARDS, lung injury, omega-3 PUFAs, DHA

## Abstract

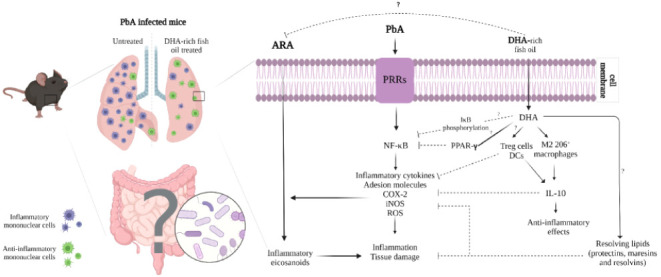

Acute lung injury (ALI) and acute respiratory distress syndrome (ARDS) are severe complications that can occur in infections caused by any *Plasmodium* species. Due to the high lethality rate and the lack of specific treatment for ALI/ARDS, studies aimed at understanding and searching for treatment strategies for such complications have been fundamental. Here, we investigated the protective role of dietary supplementation with DHA-rich fish oil against lung damage induced by *Plasmodium berghei* ANKA in a murine model. Our results demonstrated that alveolar vascular damage, lung edema, and histopathological alterations were significantly reduced in mice that received dietary supplementation compared to those that did not receive the supplementation. Furthermore, a significant reduction in the number of CD8+ T lymphocytes, in addition to reduced infiltration of inflammatory cells in the bronchoalveolar lavage fluid was also observed. High levels of IL-10, but not of TNF-α and IFN-γ, were also observed in infected mice that received the supplementation, along with a reduction in local oxidative stress. Together, the data suggest that dietary supplementation with DHA-rich fish oil in malarial endemic areas may help reduce lung damage resulting from the infection, thus preventing worsening of the condition.

Malaria has transcended centuries and remains the deadliest parasitic disease worldwide. In 2021, the number of deaths caused by this disease was around 620,000, most of which were registered in the African continent and caused by *Plasmodium falciparum*.^1^ Malaria-associated deaths generally result from several processes affecting organs, such as the brain, lungs, and kidneys, leading to cerebral malaria (CM), acute pulmonary insufficiency, and kidney failure, respectively.

While CM is a complication that mainly occurs in children infected with *P. falciparum,*(1) acute lung injury (ALI) and malaria-associated acute respiratory distress syndrome (MA-ARDS) typically occur in adults infected by any *Plasmodium* species.^2,3^ Therefore, the adherence of the parasite to the pulmonary microvasculature does not appear to be the determinant factor of lung-associated pathologies.

During malaria pathogenesis, the engagement of specific Toll-like receptors (TLRs) in the recognition of malaria antigens, such as hemozoin (Hz) and cell surface glycoproteins (GPIs), drives the gene expression of pro-inflammatory mediators, such as pro-interleukin (IL)-1β, IL-6, IL-12, IL-18, tumor necrosis factor (TNF)-α, and enzymes, including cyclooxygenase (COX)-2. The pro-inflammatory cytokines, IL-12 and IL-18, activate natural killer (NK) and CD4+ Th1 T cells that have been shown to produce interferon (IFN)-γ, which induces phagocyte activation and increase both NADPH-oxidase and inducible nitric oxide synthase (iNOS) enzyme activity, leading to the generation of microbicidal molecules, such as hydrogen peroxide (H_2_O_2_) and nitric oxide (NO), respectively. Likewise, the activation of TLR receptors promotes a series of events that result in the increased expression of intercellular and/or vascular adhesion molecules (ICAM-1 and VCAM-1), and activation of nuclear factor kappa B (NF-κB).^4^ Moreover, inflammatory monocytes and classically activated macrophages (M1), as well as CD8+ T cells, have been associated with malaria pathogenesis.^4,5^ Therefore, immune response regulation upon infection is pivotal in homeostasis maintenance and improved clinical outcomes in malaria.

A consensus exists that the nutritional status of an individual can define their susceptibility to diseases with different etiologies.^6^ This is since macro- and micronutrients acquired through a healthy diet and/or supplementation are essential to modulate and maintain the functionality of the immune system. Polyunsaturated fatty acids of the omega-3 family (Ω-3 PUFAs), such as eicosapentaenoic acid (EPA) and docosahexaenoic acid (DHA), have been extensively studied, mainly owing to their anti-inflammatory activity. Ω-3 PUFAs have been recommended in the therapy of patients with cancer, including lung cancer, due to their ability to inhibit the progression or complications of the disease.^7^ Furthermore, the enteral administration of a nutritional formula containing PUFAs to patients suffering from ALI/ARDS was reported to significantly improve oxygenation, ventilation, and lung compliance, reducing the time in the intensive care unit.^8^

DHA can reduce inflammation through several pathways^9^ as by decreasing IκB phosphorylation with consequent maintenance of NF-κB inhibition. Furthermore, DHA exerts anti-inflammatory effects through peroxisome proliferator-activated receptor-γ (PPAR-γ) activation. PPAR-γ appears to suppress the secretion of pro-inflammatory cytokines since it interferes with the translocation of NF-κB to the nucleus,^9,10^ increases the accumulation of T regulatory cells in inflammatory sites, and promotes the development of M2 macrophages.^11^ These cells produce IL-10, a cytokine that has a significant role in controlling inflammatory responses.^12^

Given that (1) malaria infection is an important cause of lung injury in adult patients; (2) ALI/MA-ARDS does not have a specific treatment; and (3) PUFAs have shown promising effects as an adjuvant treatment in lung diseases unrelated to malaria, in this study we investigated whether dietary supplementation with DHA-rich fish oil could protect the lungs from the harmful effects of malarial infection.

## Results

### DHA-Rich Fish Oil Supplementation Protects C57BL/6 Mice Against Severe Symptoms of Malaria and Controls Blood Parasitemia in the Initial Days of Infection

**Figure 1:**
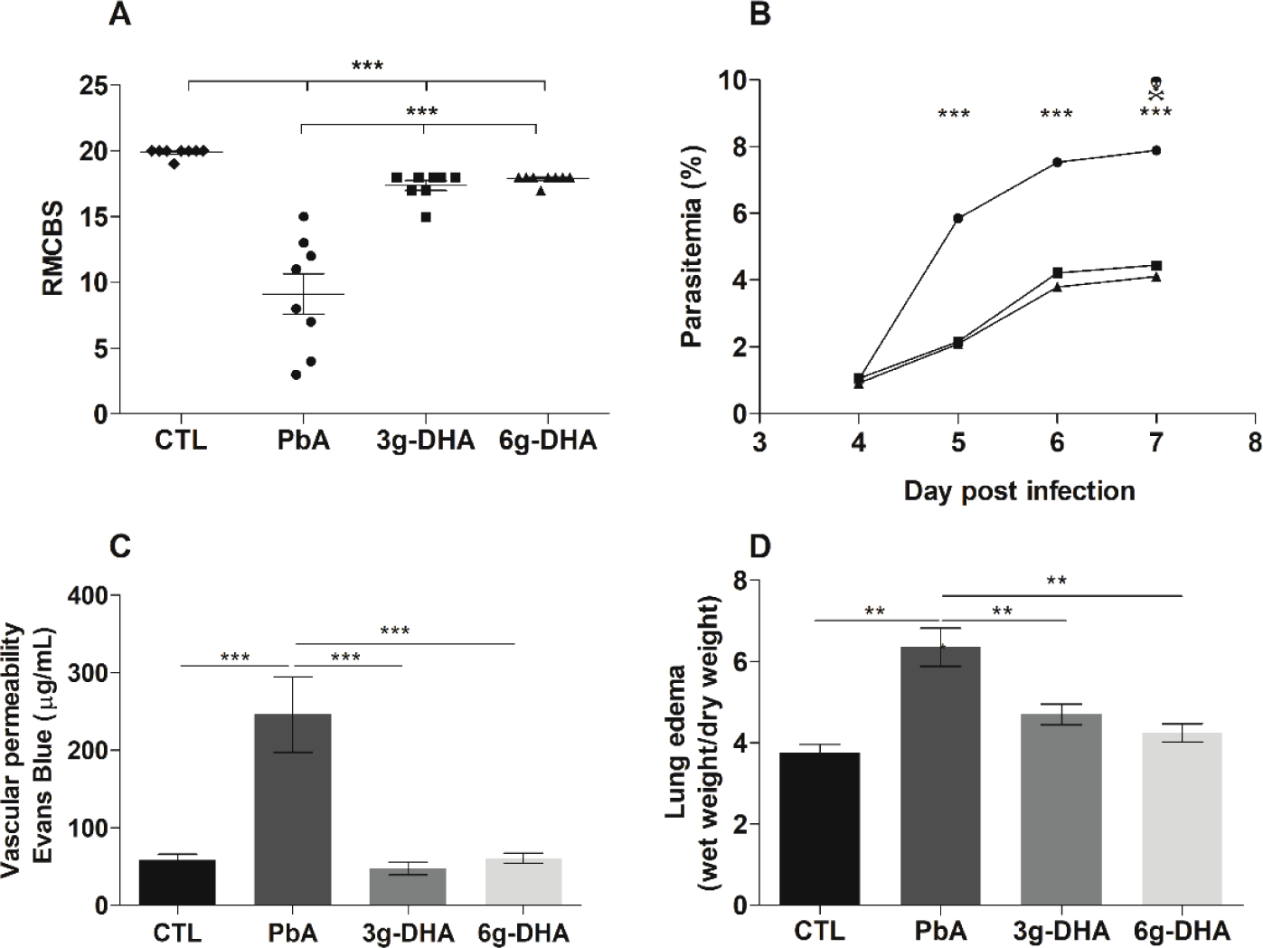
DHA-rich fish oil supplementation prevents the appearance of severe symptoms, controls the bloodstream schizogony in the initial days of infection, and prevents the alveolar vascular permeability and lung edema in *Plasmodium berghei* ANKA-infected mice. C57BL/6 mice received or did not receive DHA-rich fish oil daily for 15 days and then were infected with *P. berghei* ANKA (PbA). (A) Clinical assessment by the RMCBS throughout infection. (B) Blood parasitemia was evaluated from the 4th to 7th dpi. (C) On the 7th dpi, mice were anesthetized, and an Evans blue dye solution was injected intravenously for the determination of alveolar vascular permeability. (D) Lung edema determined by the ratio of dry weight/wet weight of the lungs on 7 dpi. The results are representative of two independent experiments (*n* = 9–10 mice/group in A and B and, *n* = 5 in C and D), and are shown as the frequency or mean ± SEM **p < 0.001; ***p < 0.0001. CTL: nonsupplemented and noninfected mice; PbA: nonsupplemented (circle), infected mice; 3 and 6 g-DHA: received supplementation with either 3 or 6 g DHA/kg body weight (square and triangle respectively).

C57BL/6 mice received daily DHA-rich fish oil supplementation for 15 days prior to infection and continuing to the seventh day after infection (dpi) with *P. berghei* ANKA (PbA) strain, at which point the animals were euthanized. Corroborating our previous study,^13^ infected mice that had received enteral supplementation presented a good clinical condition on the seventh dpi, as assessed by the RMCBS (mean scores of 17 and 18 for groups receiving 3 and 6 g DHA/kg body weight, respectively). On the other hand, in the absence of supplementation, infected mice (PbA group) showed a progressive deterioration of health, as indicated by a significant decrease in the RMCBS score (mean score, 9) (Figure 1A).

To investigate the effect of DHA-rich fish oil supplementation on blood parasitemia, we made thin smears daily from a drop of blood collected from the tip of the tail of infected animals. From the fifth to seventh dpi, the levels of circulating parasites remained significantly reduced in mice that received 3 and 6 g DHA/kg daily compared to those observed in the mice that did not receive the fish oil (Figure 1B).

### DHA-Rich Fish Oil Supplementation Protects C57BL/6 Mice Against Pulmonary Vascular Permeability, Lung Edema, and Histopathological Damage Induced by Malarial Infection

Since animals receiving DHA-rich fish oil were protected against neuronal symptoms of PbA infection (Figure 1A), we next investigated the effect of supplementation on the pulmonary environment via analyses of alveolar vascular permeability and lung edema. A reduced concentration of extravasated Evans blue dye from the tissue suggested that the lungs of animals receiving DHA-rich fish oil were protected from vascular damage induced by infection (Figure 1C). On the other hand, in the absence of supplementation, the infection induced significant vascular injuries, as evidenced by the highest concentration of dye recovered from lung tissue (Figure 1C). Similarly, lung edema occurred in animals from the PbA group, while it was absent in the infected mice that received the fish oil (Figure 1D). No differences were observed in terms of the alveolar vascular permeability or lung edema between the supplemented groups and the control group (nonsupplemented and noninfected mice).

**Figure 2:**
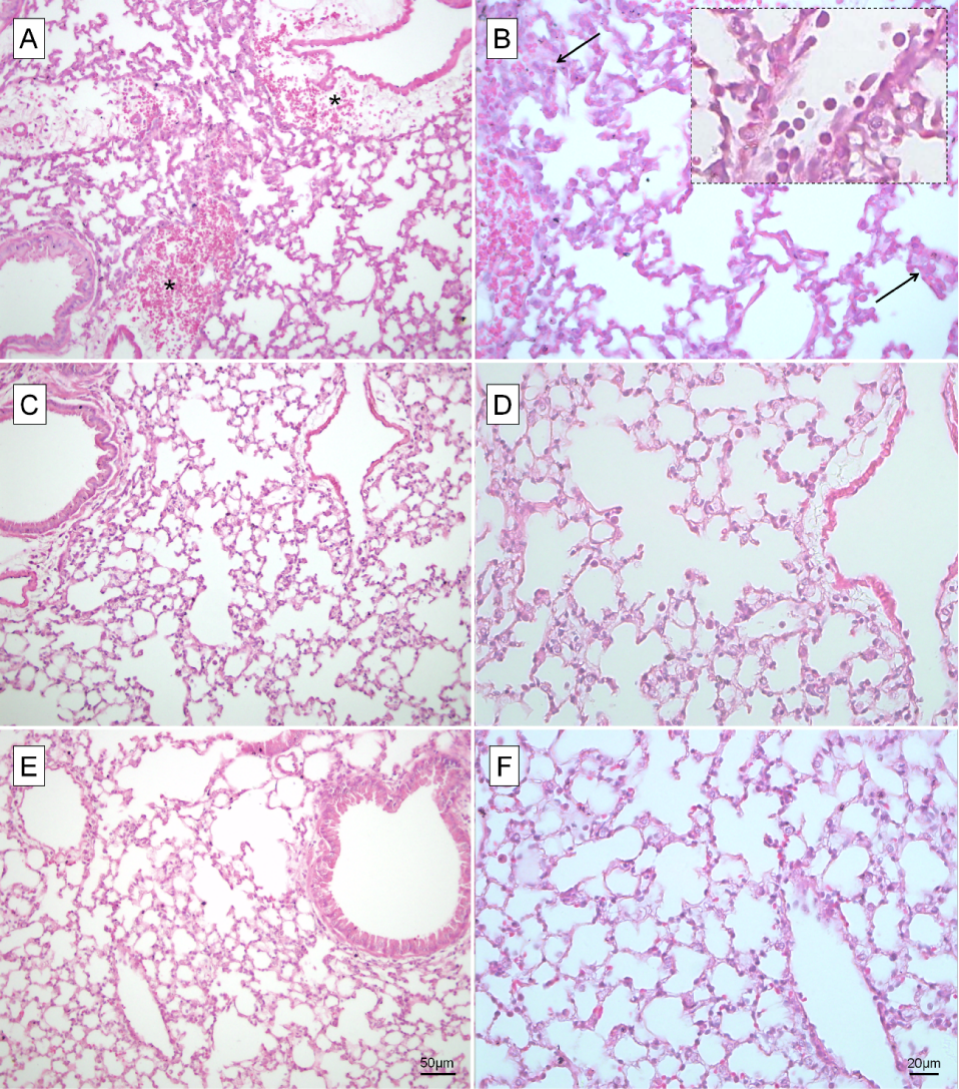
DHA-rich fish oil supplementation ameliorates the lung damage induced by 21040-360 *Plasmodium berghei* ANKA. The lungs of mice were assessed on the 7th day postinfection with *P. berghei* ANKA (PbA) strain. (A,B) Nonsupplemented PbA-infected mice showed edema and perivascular hemorrhage areas (inset) and thickening of the alveolar septa (arrow). Vascular cytoadherence of erythrocytes and polymorphonucleated cells (inset) was also observed. These alterations were mitigated in PbA-infected mice that received 3 g (C,D) and 6 g DHA/kg body weight daily (E,F). Microphotographs from a 20× objective lens; scale bar 50 μm; (A,C,E). Microphotographs from a 40× objective lens (B,D,F); scale bar 20 μm. *n* = 5/group.

The lungs of mice from the PbA group revealed intense edema and perivascular hemorrhage, the influx of inflammatory cells and vascular cytoadherence, as well as the thickening of the alveolar septa (Figure 2A,B). In comparison, infected mice that had received the fish oil supplementation, 3 g DHA/kg (Figure 2C,D) or 6 g DHA/kg (Figure 2E,F), had attenuated edema and perivascular hemorrhage, with reduced thickening of the alveolar septa. Furthermore, these groups showed a decrease in vascular cytoadherence of erythrocytes and leucocytes when compared to that of the nonsupplemented animals.

Considering the results of these analyses, where all investigated parameters were similarly ameliorated in the supplemented mice when compared to the nonsupplemented infected mice, irrespective of whether it was with the 3 or 6 g DHA/kg dose, we chose the lesser dose for the dietary supplementation of animals for the subsequent analyses.

### DHA-Rich Fish Oil Supplementation Alters the Phenotypic Profile of Lung Tissue Cells from PbA-Infected C57BL/6 Mice

**Figure 3:**
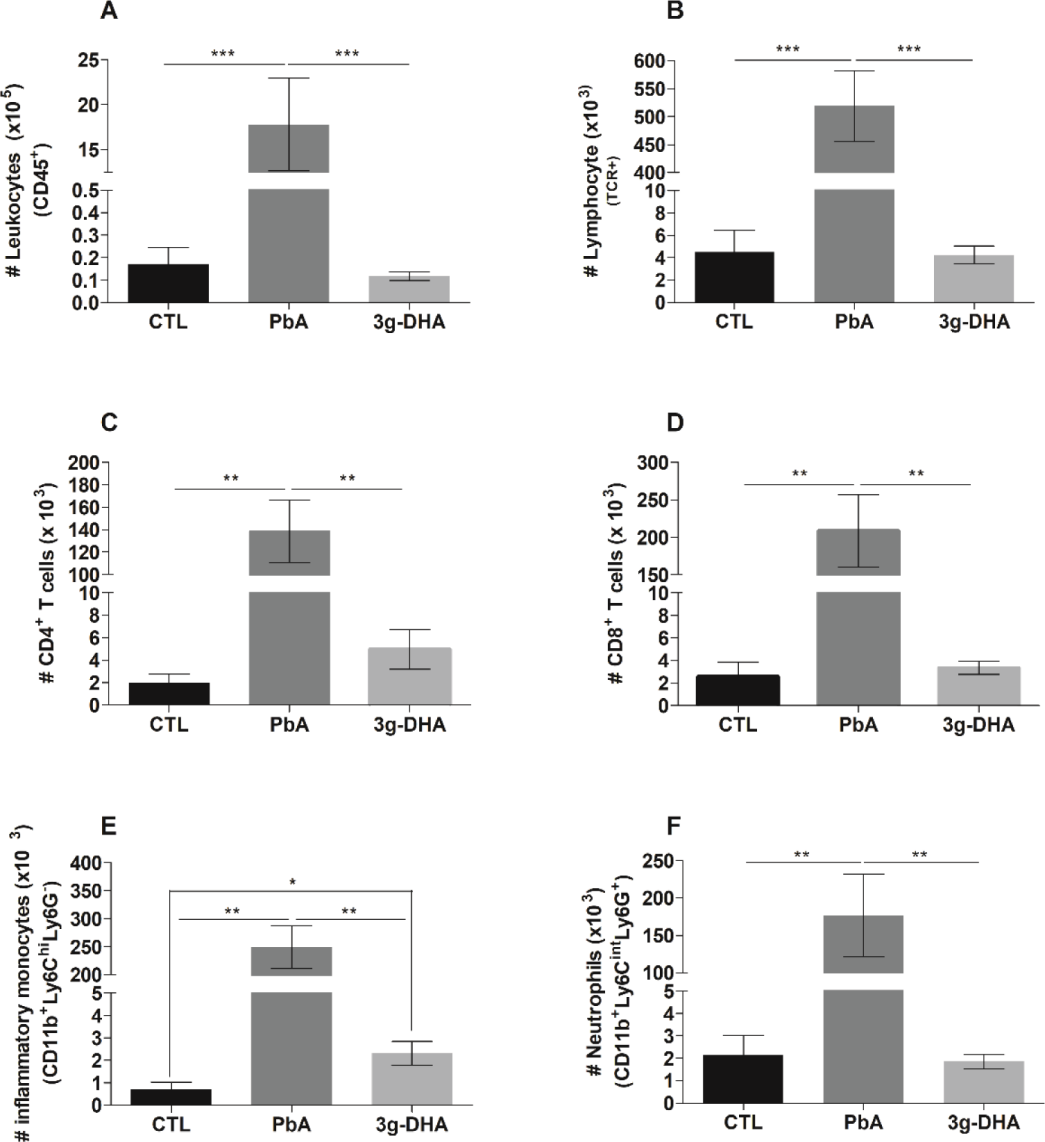
DHA-rich fish oil supplementation alters the lung cell populations in *Plasmodium berghei* ANKA-infected mice. The lungs were collected on the 7th day postinfection with *P. berghei* ANKA (PbA) strain and processed for the isolation of tissue cells. The total number of (A) leukocytes, (B) T lymphocytes, (C) CD4+ T cells, (D) CD8+ T cells, (E) inflammatory monocytes, and (F) neutrophils were analyzed by flow cytometry. The graphs show the mean number of cells expressing the specific receptor(s)/10^5^ or 10^3^ cells; *n* = 5–6 mice/group; *p < 0.01; **p < 0.001; ***p < 0.0001. CTL: nonsupplemented and noninfected mice; PbA: nonsupplemented, infected mice; 3g-DHA: received supplementation with 3 g DHA/kg body weight.

Interestingly, all cell populations investigated in this study were changed in the lung tissue of DHA-rich fish oil-supplemented animals compared with only PbA-infected mice. Considering lymphoid cells, the total numbers of leukocytes (CD45+) and T lymphocytes (TCR^+^) were significantly increased in the lungs of infected animals that did not receive supplementation, in comparison with those observed in the lungs from infected animals that received 3 g DHA/kg daily and from the control animals (Figure 3A,B). Corroborating this, the numbers of CD4+ and CD8+ T lymphocytes were found to be significantly reduced in these groups in comparison with those of the PbA group (Figure 3C,D).

Regarding the myeloid cell populations, the numbers of inflammatory monocytes (CD11b^+^Ly6C^hi^Ly6G^–^) and neutrophils (CD11b^+^Ly6C^int^Ly6G^+^) were similarly reduced in PbA-infected animals that received fish oil and control animals in comparison with those of the PbA group (Figure 3E,F).

Except for the inflammatory monocytes, which were significantly more frequent in the lungs of infected animals that had received supplementation compared to those of the control group, the rates observed for the other cell populations were similar between these groups.

### DHA-Rich Fish Oil Supplementation Reduces Cellular Infiltration in the BAL of PbA-Infected C57BL/6 Mice

**Figure 4:**
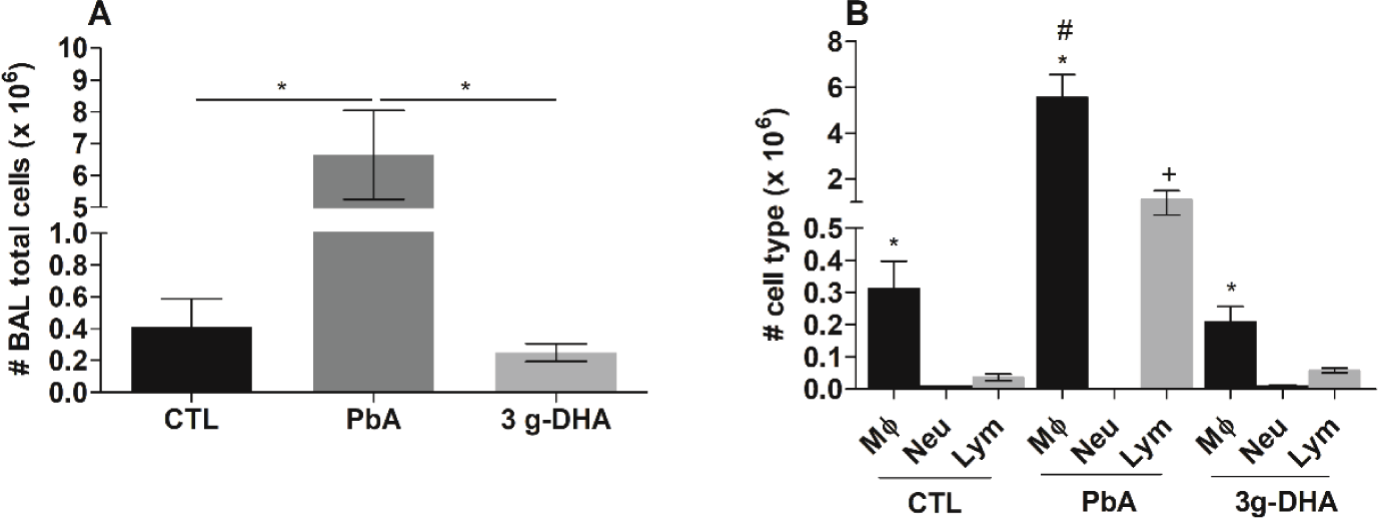
DHA-rich fish oil supplementation prevents the infiltration of inflammatory cells into the bronchoalveolar lavage fluid. On the 7th day postinfection with *P. berghei* ANKA (PbA) strain, mice were anesthetized and tracheal cannulation was performed to obtain the bronchoalveolar lavage (BAL) fluid. (A) The total number of cells (× 10^6^); *p < 0.01; and (B) the number of cells (× 10^6^) by type were determined: macrophages, neutrophils, and leukocytes. The results are shown as the frequency or the mean ± SEM; *n* = 5–6 mice/group. On B, * indicates *p* < 0.05 (MØ vs Neu and MØ vs lym within CTL, PbA, and 3g-DHA groups); # indicates *p* < 0.05 (MØ PbA vs MØ CTL and MØ PbA vs MØ 3g-DHA groups); + indicates *p* < 0.05 (Lym PbA vs Lym CTL and Lym PbA vs Lym 3g-DHA groups). CTL: nonsupplemented and noninfected mice; PbA: nonsupplemented, infected mice; 3g-DHA: received supplementation with 3 g DHA/kg body weight.

The influx of inflammatory cells to the lung tissue was also evaluated in the BAL collected from mice that received DHA-rich fish oil supplementation and those that did not. The results suggested that the supplementation protected the lungs of infected animals since they showed a significantly reduced cell infiltration in the BAL in comparison with that observed in the PbA group (Figure 4A). Furthermore, no significant difference was observed for the total number of cells in the BAL fluid from the supplemented-infected and control groups. Regarding the specific cell populations, the number of macrophages and lymphocytes was significantly increased in the PbA group compared to those of the supplemented-infected and control groups (Figure 4B). Neutrophils, on the other hand, remained absent or in lower numbers in all groups (Figure 4B).

### DHA-Rich Fish Oil Supplementation Reduces the Levels of Pro-Inflammatory Cytokines in the Lung Tissue Homogenate and Supernatant of Mediastinal Lymph Node Culture from PbA-Infected C57BL/6 Mice

**Figure 5:**
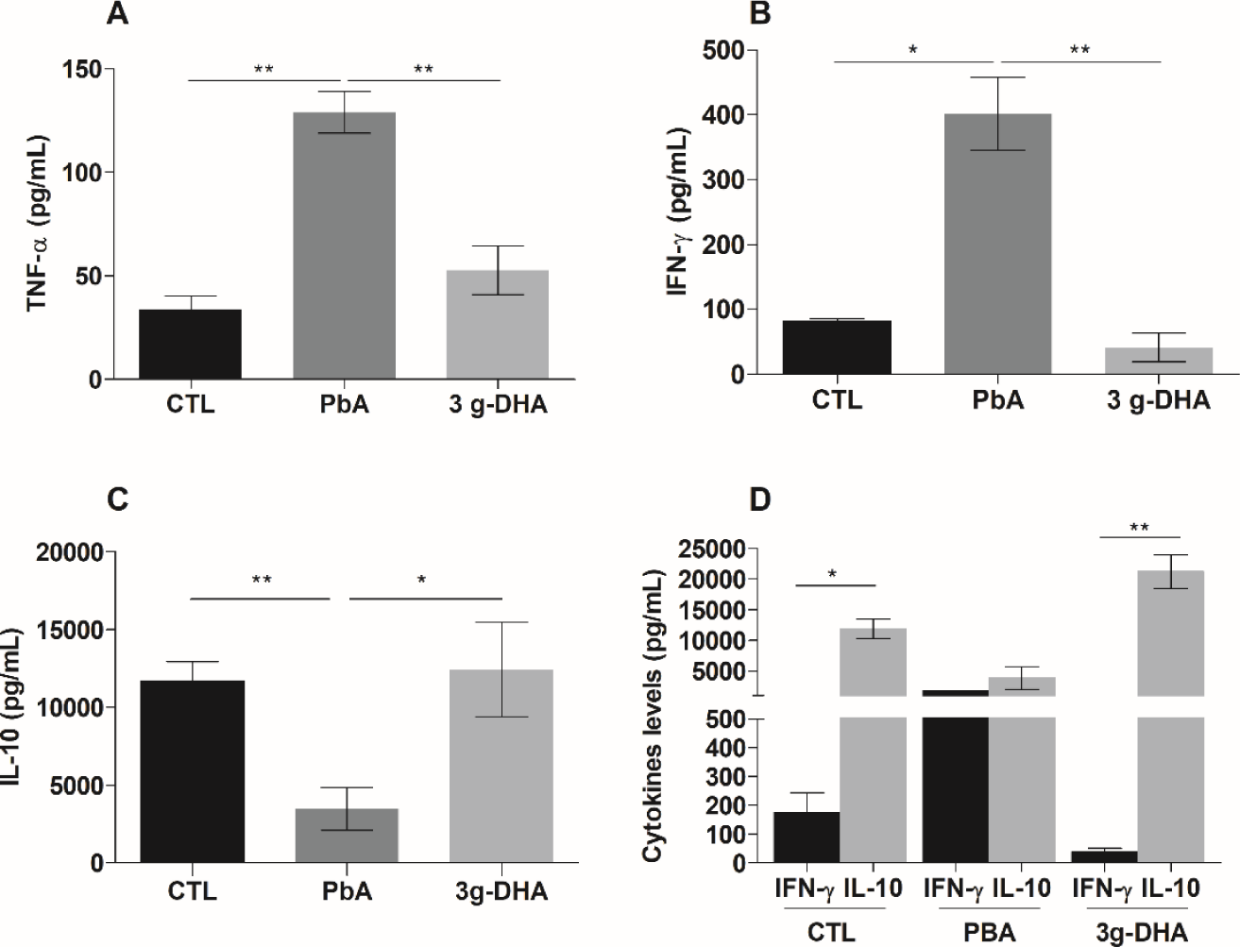
DHA-rich fish oil supplementation controls the pro-inflammatory cytokine levels in the lungs and draining lymph nodes from *Plasmodium berghei* ANKA-infected mice. On the 7th day postinfection with *P. berghei* ANKA (PbA) strain, mice were anesthetized and the lungs were removed and processed for the analysis of (A) TNF-α, (B) IFN-γ, and (C) IL-10 levels. (D) The draining lymph nodes were also removed and processed to obtain single cell suspensions, which were then stimulated with the crude malarial antigen. After 48 h, culture supernatants were collected to determine the IFN-γ and IL-10 levels. The results are representative of two independent experiments (*n* = 5 mice/group) and are shown as the means ± SEM. *p < 0.01; **p < 0.001. CTL: nonsupplement and noninfected mice; PbA: nonsupplemented, infected mice; 3g-DHA: received supplementation with 3 g DHA/kg body weight.

Considering the immunomodulatory potential of DHA, we next evaluated the pro- (IFN-γ and TNF-α) and anti-inflammatory (IL-10) cytokine profiles in lung tissue from animals. The levels of IFN-γ and TNF-α were significantly increased in the lungs of infected animals that did not receive DHA-rich fish oil supplementation, in comparison to both the supplemented and control groups (Figure 5A,B). In contrast, significantly reduced levels of IL-10 were observed only in the PbA group (Figure 5C).

Corroborating the results obtained for the tissue homogenate, IL-10 levels were also significantly higher in the culture supernatant of lymph nodes from infected mice that received supplementation and control mice that the respective IFN-γ levels (Figure 5D). In contrast, in the PbA group, the levels of both IL-10 and IFN-γ were similar (Figure 5D). Interestingly, in this group, the IFN-γ level was significantly higher than that determined in the other groups, while the IL-10 level was reduced.

### DHA-Rich Fish Oil Supplementation Controls Oxidative Stress in PbA-Infected C57BL/6 Mice

**Figure 6:**
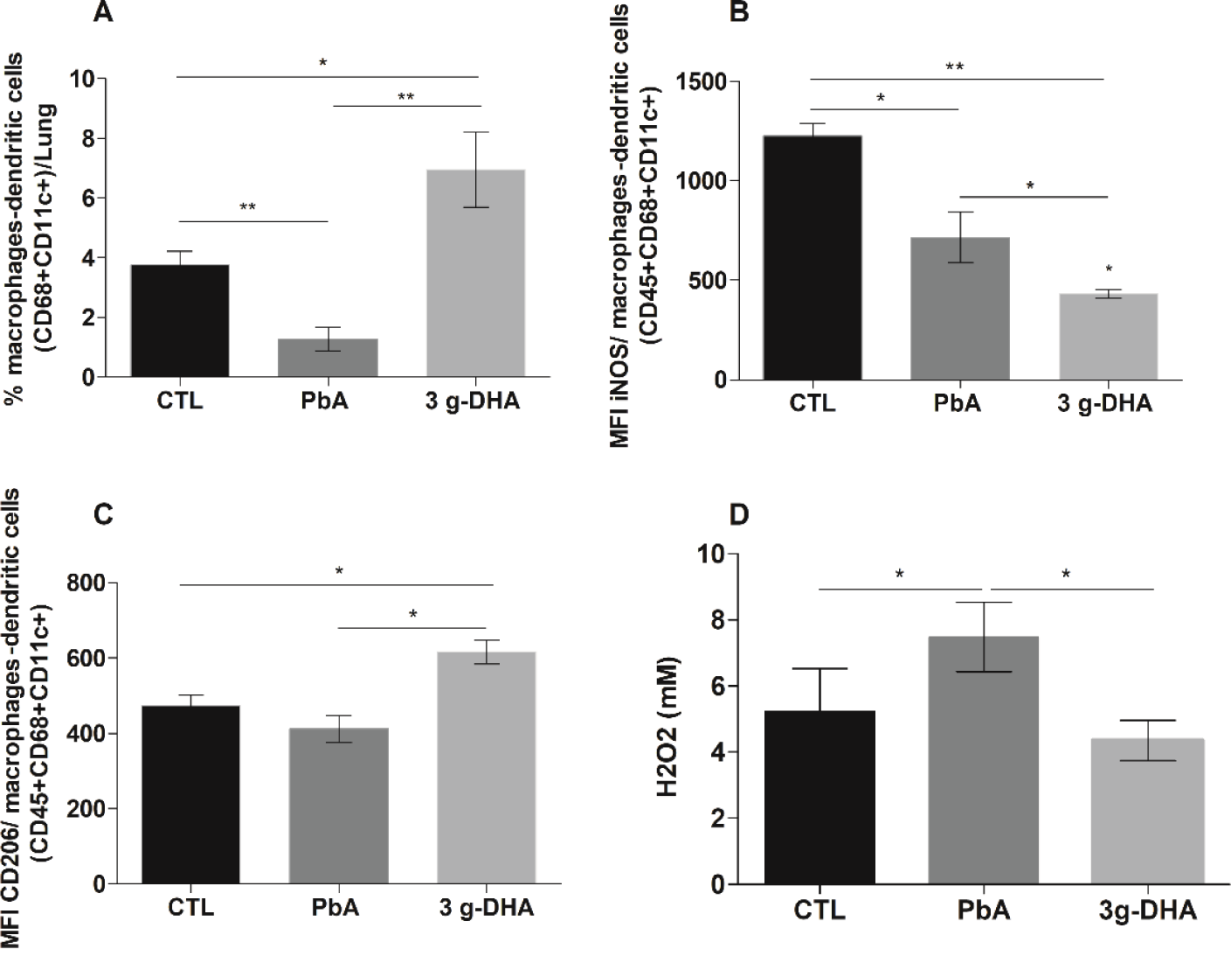
DHA-rich fish oil supplementation alters iNOS and CD206+ expression in macrophage/dendritic cells from the lung and the production of H_2_O_2_from the bronchoalveolar lavage fluid of *Plasmodium berghei* ANKA-infected mice. On the 7th day postinfection, the lung was obtained and processed to determine (A) the frequency of macrophage/dendritic cells, and (B) the median fluorescence intensity (MFI) of iNOS and (C) MFI of CD206 in this cell type. (D) The concentration of H_2_O_2_ produced by infiltrating cells in the BAL were analyzed. The results shown as the means ± SEM; *n* = 5–6 mice/group. iNOS: inducible nitric oxide synthase; CD206: mannose receptor; *p < 0.01; **p < 0.001. CTL: nonsupplement and noninfected mice; PbA: nonsupplemented, infected mice; 3g-DHA: received supplementation with 3 g DHA/kg body weight.

Considering that iNOS and CD206 are used as markers of cells with proinflammatory and anti-inflammatory profiles, respectively, we next investigated their expression in the CD45+, CD68+, and CD11c+ cells (macrophages/DCs). In infected animals, the dietary supplementation with DHA-rich fish oil-induced the expansion of this cell population in the lungs (Figure 6A). Interestingly, however, when analyzing the expression of iNOS, based on the median fluorescence intensity (MFI), it was noticed that despite the higher frequency of CD68+ and CD11c+ cells, this group had the lowest iNOS expression, when compared to that of the control and PbA groups (Figure 6B). However, an increase in CD206 expression (suggestive marker of M2 macrophages) was also observed in this group in comparison with the PbA and control groups (Figure 6C).

In the BAL, oxidative stress was analyzed through the release of H_2_O_2_, which was significantly increased in the PbA group when compared to that of the supplemented and control groups (Figure 6D).

## Discussion

In this study, we show that enteral supplementation with DHA-rich fish oil prevented the occurrence of alveolar permeability and, consequently, lung edema observed in PbA-infected C57BL/6 mice at 7th dpi. In these animals, the parasite load in the bloodstream was also significantly reduced from fifth to seventh dpi. However, it remains to be investigated whether supplementation with DHA-rich fish oil can reduce the number of parasites sequestered in the lungs. It is important to emphasize, however, that despite discussions regarding the adherence of iRBCs in the lung microvasculature as being an essential factor associated with human pulmonary injury, ALI/ARDS can also occur during infections caused by *P. vivax*, *P. knowlesi*, and *P. ovale*,^2,14−16^ where cytoadherence is not completely understood. In the same way, in the malarial murine model, ALI/MA-ARDS occurs in at least 90% of C57BL/6 mice infected with *P. berghei* NK65,^17^ even though parasite sequestration on microvessels is not expressive. Therefore, the harmful effects of malaria infection in the lung cannot be restricted to the sequestration of parasites, but may involve local and systemic inflammatory responses induced by malarial antigens, such as Hz.^18^ In our study, although it was not possible to investigate the mechanical lung impairment in the mice,^19^ lung edema and histopathological changes, common signs of life-threatening respiratory distress in infected humans and mice, were improved in PbA-infected mice receiving DHA-rich fish oil.

Regarding the influx of inflammatory cells into the lung, we observed that PbA infection led to inflammatory infiltrate rich in mononuclear cells (with a predominance of monocytes and lymphocytes), while DHA-rich fish oil supplementation significantly reduced the number of these cells in the lung tissue, as well as in the BAL fluid. A previous study reported that during PbA infection in C57BL/6 mice, ALI occurs followed by the recruitment of monocytes, which remained in the lung tissue as macrophages.^5,20^ Activated macrophages produce many oxidative metabolites and pro-inflammatory cytokines that contribute to cellular damage in the lung parenchyma. During malaria infection, several antigens, including Hz, can activate monocytes/macrophages via the inflammasome pathway, leading to M1 polarization, which is associated with the production of pro-inflammatory cytokines.^3,21,22^ It is known that one of the main microbicidal mechanisms of activated macrophages in the inflammatory infiltrate is the production of reactive oxygen species (ROS) and nitrogen species (RNS), such as H_2_O_2_ and NO, respectively. The production of these molecules constitutes the oxidative burst, important for parasite killing, but also one of the main causes of oxidative damage to cell membranes. In our study, we found that PbA infection increased the level of production of H_2_O_2_ from cells in the BAL fluid. At the same time, DHA-rich fish oil supplementation appears to reduce the levels of these reactive species, which may have contributed to the decrease in pulmonary tissue damage.

Regarding the lymphocyte populations, we found that DHA-rich fish oil supplementation significantly reduced the number of CD8+ T lymphocytes in the lung tissue of infected mice. Previous studies using the same model of malaria infection showed that the recruitment of CD8+ T cells to the lung was a determinant of injury since treatment with anti-CD8 antibodies was able to prevent pulmonary vascular leakage.^5,23^ CD8+ T cells contribute to lung injury through the secretion of IFN-γ, granzyme B, and/or perforin, which can activate endothelial cells and/or disrupt tight junctions in the pulmonary epithelium,^5^ with a consequent increase in vascular permeability and edema. Interestingly, a significant reduction in the frequency of CD8+ T cells in the spleen associated with DHA-rich fish oil supplementation was previously shown by our group.^13^

The balance between the pro- and anti-inflammatory cytokines is crucial for controlling infection and re-establishing homeostasis.^24^ In this study, we observed that PbA infection increased the levels of TNF-α and IFN-γ in the lung tissue and draining lymph nodes. These results are in line with those shown previously since the largest source of these cytokines is activated macrophages and lymphocytes. Previous studies have already associated the severity of malaria with pro-inflammatory cytokine production and secretion, both in humans^25^ and in murine experimental models.^26^ Pro-inflammatory cytokines are associated with increased expression of adhesion molecules on endothelial cells, which are responsible for leukocytes and iRBCs sequestration, causing obstruction of the microvasculature and inducing diapedesis of leukocytes into the lung tissue.^27^ On the other hand, increased IL-10 levels were observed in the lungs and draining lymph nodes of infected mice that received DHA-rich fish oil when compared to nonsupplemented infected mice. These data hold significance, as a transition from a pro-inflammatory response in the acute phases of infection toward a persistent anti-inflammatory profile has been reported, and this appears to be associated with a lower frequency of severe malaria in endemic regions.^28^ IL-10 has been shown to downregulate the major histocompatibility complex (MHC) II, thus suppressing antigen presentation, T cell activation capacities of DCs and macrophages, and to suppress NF-κB pathway, thereby inhibiting inflammatory cytokine (IL-1α, IL-1β, IL-6, IL-12, IL-18, GM-CSF, G-CSF, M-CSF, and TNF-α) and chemokine production (iMCP-1, MCP-5, RANTES, MDC, IL-8, IP-10, and MIP-2), and costimulatory molecule expression (CD80 and CD86).^29,30^ IL-10 can also act directly on T cells, blocking proliferation and pro-inflammatory cytokine production.^31^ Hence, IL-10 can directly impact both innate and adaptive immune responses. In our model, the increased IL-10 levels in mice that received DHA-rich fish oil supplementation may have been reflected in the reduced production of the pro-inflammatory cytokines, TNF-α and IFN-γ, as well as the decrease in the influx of inflammatory cells into lung tissue.

In mouse models of *P. chabaudi* infection, several different immune cells, such as CD4+ T, CD19+ B, and CD11c^high^ cells, were identified as sources of IL-10.^32^ In addition, in humans infected with *P. falciparum*, CD4+ and CD8+ T cells obtained from peripheral blood, monocytes, and monocyte-derived DCs were all able to produce IL-10.^33,34^ These data corroborate our findings, since we found increased levels of IL-10 in the draining lymph nodes of infected mice that received DHA-rich fish oil supplementation, and the lymph nodes comprise a large concentration of activated T lymphocytes.

In our model, we found evidence that DHA can still act through the PPAR-γ pathway since mice receiving DHA-rich fish oil show decreased pro-inflammatory cytokine and H_2_O_2_ production, as well as reduced iNOS expression. In addition, there is an increased number of CD206+ cells, indicating possible polarization toward the M2 macrophage profile in the lungs.^35,36^ Previous studies have shown that activated macrophages express significant PPAR-γ levels,^37,38^ whereas, in the absence of PPAR-γ signaling, these cells tend to produce pro-inflammatory cytokines.^39^ Moreover, when activated, PPAR-γ induces a phenotypic polarization of macrophages from M1 to M2 at inflamed sites with consequent production of anti-inflammatory cytokines. These M2 macrophages have inflammation-resolving functions, such as the production of protectins, maresins, and resolvins, endogenous lipid mediators biosynthesized from Ω-PUFA DHA with pro-resolving effects.^40−42^ Indeed, previous studies showed that pretreatment of mice with resolvins and maresins was able to reduce the levels of pro-inflammatory cytokines and neutrophil migration in BALF as well as attenuate inflammation in lung tissue in a murine model of LPS-induced ALI through a process involving the PPAR-γ/NF-κB pathway.^36,43^ During the infection with *Trypanosoma cruzi*, PPAR-γ pathway activation using agonists also leads to inhibition of pro-inflammatory cytokines associated with the M2 macrophage polarization and inhibition of the NF-κB pathway.^44^

The discussion on how diet impacts inflammatory diseases is a topical issue. It is known that the ratio of Ω-6/ Ω-3 PUFAs in the modern Western diet is unbalanced owing to a predominance of foods rich in Ω-6.^45^ Ω-6 PUFAs typically upregulate inflammation by acting as precursors to arachidonic acid-derived pro-inflammatory eicosanoids, while the Ω-3 PUFA, DHA, resolves inflammation by competing within the same enzymatic pathway and producing resolving lipids that have anti-inflammatory effects, such as decreased adhesion molecule expression on the surface of monocytes^9,46^ and reduction of pro-inflammatory cytokine production.^41^ Ω-6 and Ω-3 PUFAs also appear to have opposing effects on intestinal homeostasis. We hypothesize that DHA-rich fish oil decreases inflammatory responses through IL-10 upregulation or via ARA inhibition, inducing the secretion of resolving lipids or blocking the NF-κB pathway, as indicated in other works.^9^ Nevertheless, data from mouse models suggest that oral administration of Ω-3 PUFAs is also associated with composition changes in intestinal microbiota.^47^ Moreover, Robertson and collaborators have reported that Ω-3 supplementation induces a reversible increase in several short-chain fatty acid-producing bacteria, including butyrate-producing prokaryotes.^47^ In this regard, high doses of DHA-rich fish oil may alter the gut microbiota and indirectly affect the host’s immune response upon PbA infection, since changes in the intestinal microbiome can interfere with the inflammation state.^48^ However, further studies are required to investigate the effect of DHA-rich fish oil supplementation on gut microbiota and its repercussions on the severity of malaria.

## Conclusion

In summary, our data suggest that dietary supplementation with DHA-rich fish oil may protect the lungs against the harmful effects of malarial infection, which can result in ALI/MA-ARDS. However, the same points need attention: (1) Although the daily dose of 3 g DHA/kg body weight tested in the present study may be considered impracticable for use in humans, studies conducted with lower doses focused on the parasitic load, despite other important issues involved in the context of severe malarial infection. (2) Although the parasitological and histopathological findings of the PbA group suggest a poor prognosis of progressive pulmonary complications, in this study the mechanical lung impairment could not be evaluated. (3) Finally, as lung injury associated with ALI/MA-ARDS can occur independently of the *Plasmodium* species, we cannot state that the attenuation of the harmful effects of the infection observed in the lungs of animals infected with PbA can be extrapolated to other species with different biological characteristics.

## Material and Methods

### Mice, Infection, and Treatment

Six-to-eight-week-old female C57BL/6 mice were obtained from the Reproduction Biology Center at the Federal University of Juiz de Fora/UFJF, Minas Gerais, Brazil. The mice were housed in the animal facilities at the Parasitological Research Nucleus, with daily veterinary monitoring for welfare conditions and humane end point intervention, with food and water *ad libitum*.

Mice were supplemented with DHA-rich fish oil daily as previously described.^13^ Briefly, 200 μL of fish oil (Essential Nutrition) containing 3 or 6 g of DHA/kg of body weight was administered to each animal by gavage. Doses were defined based on a previous study where 60 and 80% of animals, respectively, were protected against CM following infection.^13^ Fifteen days after the start of enteral supplementation, mice were infected with 10^5^ infected red blood cells (iRBCs) by the *P. berghei* ANKA (PbA) strain via the intraperitoneal route (i.p.). The supplementation was maintained until the seventh day postinfection (dpi) when mice were euthanized. Nonsupplemented groups of noninfected mice (control group) and infected mice (PbA group) were also used.

### Clinical Assessment and Parasitemia

After the infection, mice were clinically monitored according to an established protocol, the “Rapid Murine Coma and Behaviour Scale” (RMCBS), as previously described.^49^ Animals showing clinical scores = 20 were completely healthy, while those with clinical scores ≤ 8 were euthanized after anesthesia (100 mg/kg ketamine and 10 mg/kg xylazine).

To determine the parasitemia load, thin blood smears (TBS) were prepared daily with a drop of blood collected from the tail vein. The smears were naturally dried, fixed with methanol, and stained with Giemsa. The percentage of parasitemia was determined by microscopic analysis considering the total number of iRBCs in a total of 1,000 erythrocytes.

### Alveolar Vascular Integrity

Evans blue staining was used to assess pulmonary vascular integrity since this dye crosses the vascular endothelium only when associated with serum albumin. Briefly, on the seventh dpi, mice were anesthetized, and then a solution of 2% Evans Blue dye (Sigma-Aldrich) was injected intravenously via the lateral tail vein (i.v.; 200 μL/animal). After 45 min, animals were euthanized and perfused with 10 mL of phosphate-buffered saline (1× PBS). Lungs were collected and submerged in a 10% formamide solution for 48 h at 37 °C. The Evans blue dye concentration recovered from pulmonary tissue was determined using a spectrophotometer at 620 nm. The absorbance was normalized according to the lung tissue weight of each animal.

### Lung Edema

Lung edema was determined as described previously.^50^ After euthanasia, the lungs were carefully removed from the thoracic cavity and immediately weighed to assess the wet weight (WW). The lungs were then dried for 48 h at 60 °C and weighed to assess the dry weight (DW). The water content of the lungs was calculated, and the values were expressed as the ratio between DW/WW.

### Lung Histopathology

After euthanasia, the lungs were collected and fixed in 4% buffered formaldehyde. The lungs were processed according to routine histological procedures. Briefly, each tissue sample was dehydrated in alcohol, diaphonized in xilol, and embedded in paraffin. Paraffin blocks were sectioned at 5 μm and stained with hematoxylin and eosin (H and E) then observed under a light microscope.

### Cell Isolation from the Lungs

Lungs were harvested, washed with 1× PBS, and placed in Petri dishes with RPMI 1640 medium (Sigma-Aldrich). The whole lung was cut into approximately 1 mm-sized pieces with scissors, incubated with 2 mL of a digestion medium [RPMI 1640 medium supplemented with 100 U/mL penicillin (Sigma-Aldrich), 100 μg/mL streptomycin (Sigma-Aldrich), and 128 μg/mL collagenase I (Roche)] and kept at 37 °C for 1 h. Tissue digestion was stopped by adding 500 μL of RPMI medium supplemented with 5% heat-inactivated fetal bovine serum (FBS; Life Technologies) and 0.01% DNase (Sigma-Aldrich). Following this, the tissue was macerated through a cell strainer (70 μm, BD Biosciences) by using a syringe plunger. The cell suspensions were then centrifuged at 10 °C and 300 × *g* for 10 min and the pellets were resuspended in FACS buffer (1× PBS with 2% FBS) for further staining.

### Phenotyping of Lung Cells

For phenotyping, the lung cells were transferred to 96-well V-bottom plates and centrifuged at 10 °C and 300 × *g* for 10 min. The supernatants were discarded, and the cells were resuspended in 50 μL of cell viability marker (LIVE/DEAD Fixable Violet Dead Cell Stain kit, Invitrogen), and diluted according to the manufacturer’s recommendations. After incubation at 4 °C for 30 min, the cells were washed with 100 μL of 1× PBS containing 5% FBS. After another centrifugation for 10 min at 500 × *g*, the cells were incubated for 30 min at 4 °C with 50 μL of FACS buffer containing the following antibodies: anti-Fcγ III/II (CD16/32) receptor (2.4G2; BD Biosciences), antimouse TCR PE (H57–597, BD Pharmingen), antimouse CD45 FITC (30F11, BD Pharmingen), antimouse CD11b PerCP-Cy5.5 (M1/70, BD Pharmingen), antimouse Ly6C PE-Cy7 (AL-21, BD Pharmingen), antimouse CD206 PE-Cy7 (MR6F3, Invitrogen), antimouse Ly6G APC (1A8; BD Pharmingen), antimouse F4/80 PE-Texas Red (BM8; Invitrogen), antimouse CD11c PerCP-Cy5.5 (HL3; BD Pharmingen), antimouse CD4 APC-H7 (GH1.5, BD Pharmingen), or antimouse CD8 AF700 (53-6.7; BD Pharmingen). For the detection of intracellular markers in some analyses, cells were fixed and permeabilized using a commercial kit (BD Biosciences) and subsequently incubated with a mix of antibodies (antimouse CD68 eFluor 660, FA-11, Invitrogen; and antimouse iNOS APC-eFluor 780, CXNFT, eBioscience) diluted in permeabilization buffer (Perm/Wash, BD Biosciences) for 40 min at 4 °C. After unbound antibodies were removed by washing, the cells were resuspended in 200 μL of PBS and kept on ice until analysis.

Fluorescence-minus-one (FMO) controls were used to establish the analysis gates. In the gate strategy (Figure S1), a region was defined based on forward (FSC) and side (SSC) scatter profiles. Dead cells were excluded based on live/dead labeling, and then the single cells were selected on a dot plot created to exclude cell aggregates: FSC-H vs FSC-A. Cell populations of interest were then set as follows: total leukocytes: CD45+; total T lymphocytes: TCR^+^; CD4 T lymphocytes: CD3+ CD4+; CD8 T lymphocytes: CD3+ CD8+; inflammatory monocytes: CD11b^+^Ly6C^hi^Ly6G^–^; neutrophils: CD11b^+^Ly6C^int^Ly6G^+^; macrophages/DCs: CD45+ CD68+ CD11c+. At least 300,000 events were acquired per sample on a CytoFlex flow cytometer (Beckman Coulter), and data were analyzed using FlowJo software (version 10.0; Figure S1).

### Bronchoalveolar Lavage (BAL)

Mice from all experimental groups were anesthetized on the seventh dpi. Next, the trachea was exposed through a midline incision and cannulated with a sterile 21-gauge T catheter (BD Bioscience). BAL was performed by instillation and aspiration of 1 mL of 1× PBS into the lungs. The retrieved BAL fluid (∼0.8 mL) was used for the determination of total and differential cell counts. For this, the BAL fluid was centrifuged at 300 × *g* for 5 min, and the supernatant was discarded. The pellet was suspended in 200 μL of 1× PBS for the total leukocyte count in a Neubauer chamber. Meanwhile, 4 × 10^4^ cells/mL were used for the preparation of slides using the cytospin technique (Cytospin, ThermoFisher). The cells were stained with Giemsa solution, and the differential cell count was performed to verify the percentage of cell populations of interest (macrophage, lymphocytes, and neutrophils) under optical microscopy (100× objective).

### Cytokine Quantification

Pro- and anti-inflammatory cytokines were measured in the lung homogenates and culture supernatants of cells from the mediastinal lymph nodes. Lung tissue homogenates were obtained using a cytokine extraction solution [0.4 M NaCl, 0.05% Tween-20, 0.5% bovine serum albumin, 0.1 mM phenylmethylsulfonyl fluoride (Sigma-Aldrich), 0.1 mM benzethonium chloride (Sigma-Aldrich), 10 mM EDTA,and 0.02% aprotinin (Sigma-Aldrich)], according to a protocol described previously.^13^

To obtain lymph node cell culture supernatants, 1 × 10^6^ cells/well from mediastinal lymph nodes were plated in a 96-well plate and stimulated with 50 μg/mL of PbA crude antigen. After 48 h, the supernatants were collected and stored at −80 °C until use.

Cytokine levels (IL-10, IFN-γ, and/or TNF-α) were determined using the OptEIA kits following the manufacturer’s instructions (BD Biosciences) and analyzed by spectrophotometry at 450 nm. The concentration of each cytokine in the samples was determined from the linear regression equation, which was generated from the absorbance values of the respective standard curve.

### H_2_O_2_ Dosage

To determine H_2_O_2_ release, a horseradish peroxidase (HRP)-dependent phenol red oxidation microassay was used. Briefly, 2 × 10^5^ cells collected from BAL were suspended in 1 mL of freshly prepared phenol red solution consisting of ice-cold Dulbecco’s PBS containing 5.5 mM dextrose, 0.56 mM phenol red (Sigma-Aldrich), and 8.5 U/mL HRP type II (Sigma-Aldrich). Following this, 100 μL of each cell suspension was added to each well of a 96-well plate and incubated for 1 h at 37 °C in a humid atmosphere containing 5% CO_2_ with 10 μL of phorbol myristate acetate (PMA; 1 μg/mL, Sigma-Aldrich). The reaction was stopped with 10 μL of 1 N NaOH. The absorbance was measured at 620 nm in a microplate reader (MR 5000, Dynatech Laboratories Inc.). Conversion of absorbance to μM H_2_O_2_ was done by comparison to a standard curve generated with known concentrations of H_2_O_2_ (5–40 μM), as described previously.^13^

### Statistical Analysis

Statistical analyses were performed in GraphPad Prism (GraphPad software, version 6.0). A normality test (Shapiro–Wilk test) was carried out to assess the distribution of the variables. If the distribution was parametric, a one-way analysis of variance (ANOVA) test was performed with a Tukey’s test for multiple comparisons. If the distribution was not parametric, then the Mann–Whitney U test was performed to compare the means between the two groups. Values of *p* < 0.05 were considered statistically significant.

## Supporting Information Available

Representative gating strategy to identify the different cell populations in the lung (PDF)

## Notes

All experimental procedures with animals were approved by the Ethics Committee on Animal Research of UFJF (protocol number # 06/2022).

## Abbreviations

ALI: acute lung injury
ARDS: acute respiratory distress syndrome
bal: bronchoalveolar lavage fluid
IL-10: interleukin 10
TNF-α: tumor necrosis factor-alfa;
IFN-γ: interferon-gama;
DHA: docosahexaenoic acid;
TLR: Toll-like receptors
Hz: hemozoin
GPI: cell surface glycoprotein
IL-1β: interleukin 1β
COX2: cyclooxygenase 2
NK: natural killer
iNOS: inducible nitric oxide synthase enzyme
H_2_O_2_: hydrogen peroxide
NO: nitric oxide
ICAM-1: intercellular adhesion molecule-1
VCAM-1: vascular cell adhesion molecule-1
NF-κB: nuclear factor kappa B
EPA: eicosapentaenoic acid
PPAR-γ: peroxisome proliferator-activated receptor- γ
MIF: median fluorescence intensity
CTL: control
ROS: reactive oxygen species
RNS: nitrogen species
MHCII: major histocompatibility complex II
DC: dendritic cell
MØ: macrophages
RBMC: rapid murine coma and behavior scale
TBS: thin blood smears
dpi: day postinfection
WW: wet weight
DW: dry weight
FBS: fetal bovine serum
