# Supplementary material for: Lung Damage Induced by Plasmodium berghei ANKA in Murine Model of Malarial Infection is Mitigated by Dietary Supplementation with DHA-Rich Omega-3
Source: ACS Infect Dis. 2024 Sep 20;10(10):3607–17. doi: 10.1021/acsinfecdis.4c00482 (PMC11474944; doi:10.1021/acsinfecdis.4c00482)
Supplement: Supplementary file 1 — id4c00482_si_001.pdf [file id4c00482_si_001.pdf]

Supporting Information

LUNG DAMAGE INDUCED BY *Plasmodium berghei* ANKA IN MURINE MODEL OF MALARIAL INFECTION IS MITIGATED BY DIETARY SUPPLEMENTATION WITH DHA-RICH OMEGA-3

Affiliations: Affiliations: Carolina David-Vieira[¶], Barbara Albuquerque Carpinter[¶], Jéssica Correia Bezerra-Bellei[¶], Letícia Ferreira Machado[¶], Felipe Oliveira Raimundo[¶], Cinthia Magalhães Rodolphi[¶], Daniela Chaves Renhe[¶], Isabella Rodrigues Nogueira Guedes[¶], Fernanda Mikaela Moreira Gonçalves[¶], Ludmila Ponce Monken Custódio Pereira[¶], Marcos Vinicius Rangel Ferreira[§], Haroldo Lobo dos Santos Nascimento[#], Adolfo Firmino Neto[#], Flávia Lima Ribeiro-Gomes[§], Vinicius Novaes Rocha[#], Juciane Maria de Andrade Castro[¶], Kézia Katiani Gorza Scopel[¶]*

[¶] Research Centre of Parasitology. Department of Parasitology, Microbiology and Immunology and Post-Graduate Program in Biological Science, Federal University of Juiz de Fora, Juiz de Fora, Brazil. 36036-900

[§] Laboratory of Malaria Research. Oswaldo Cruz Institute, Fiocruz, Rio de Janeiro, Brazil. 21040-360

[#] Research Centre of Pathology and Veterinary Histology. Department of Veterinary Medicine, Federal University of Juiz de Fora, Juiz de Fora, Brazil. 36036-900

*Corresponding author: Kézia K.G. Scopel (keziagscopel@gmail.com/kezia.scopel@ufjfj.br)

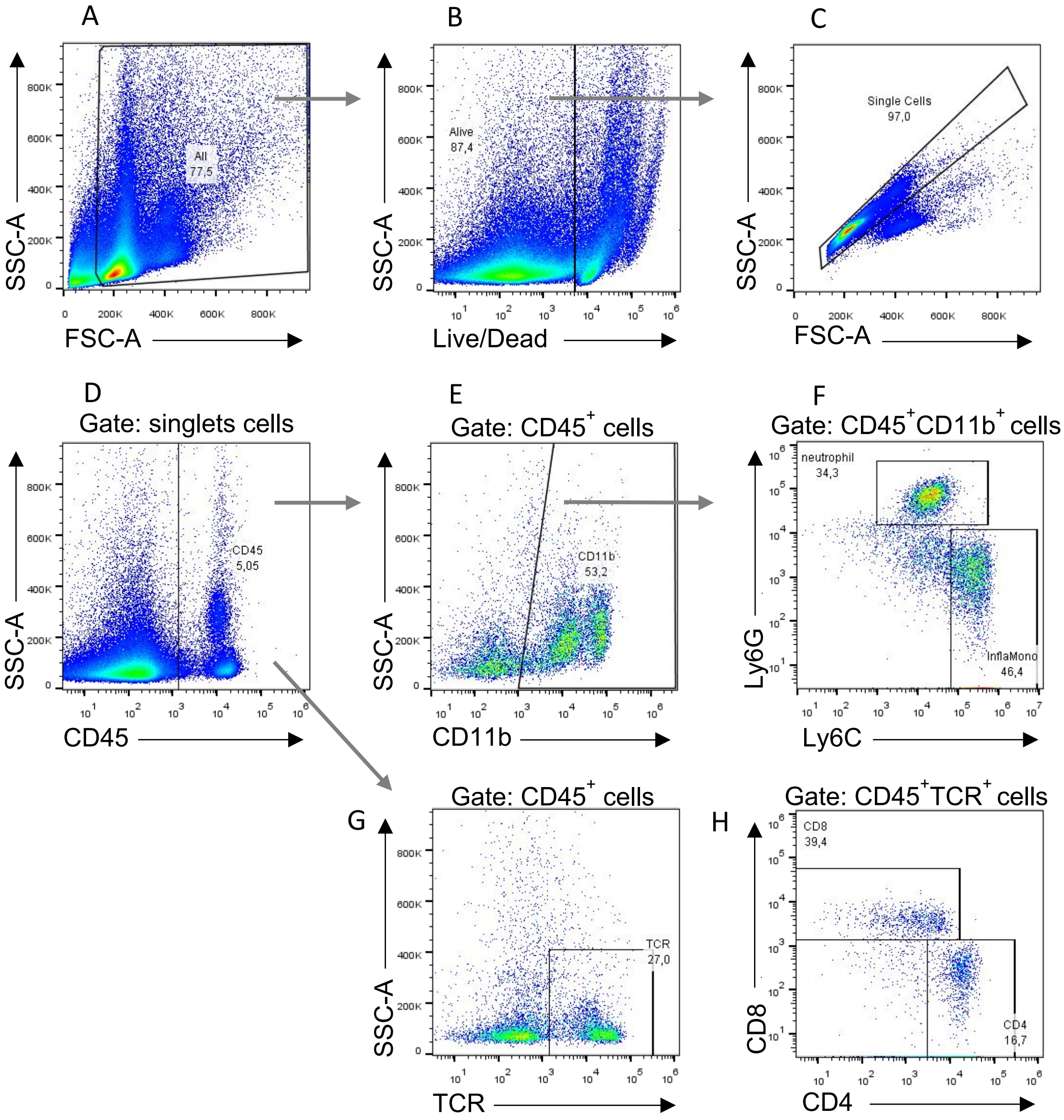

Figure S1 Representative gating strategy to identify the different cell populations in the lung. After excluding debris (A), dead cells (B) and duplets (C), the (D) leukocytes ($CD45^{+}$), (E) myeloid cells ($CD11b^{+}$), (F) neutrophils ($CD11b^{+}Ly6G^{+}Ly6C^{int}$) and inflammatory monocytes ($CD11b^{+}Ly6G^{-}Ly6C^{hi}$), (G) T cells ($TCR^{+}$), (H) CD4 T cells ($TCR^{+}CD4^{+}$) and CD8 T cells ($TCR^{+}CD8^{+}$) were identified based on the expression of the markers described in the Material and Methods section.
